# Clinical and Radiological Features of *Pneumocystis jirovecii* Pneumonia in Children: A Case Series

**DOI:** 10.3390/jof10040276

**Published:** 2024-04-09

**Authors:** Erica Ricci, Claudia Bartalucci, Chiara Russo, Marcello Mariani, Carolina Saffioti, Erika Massaccesi, Filomena Pierri, Giacomo Brisca, Andrea Moscatelli, Roberta Caorsi, Bianca Bruzzone, Maria Beatrice Damasio, Anna Marchese, Alessio Mesini, Elio Castagnola

**Affiliations:** 1 Division of Infectious Diseases, IRCCS Istituto Giannina Gaslini, Via Gerolamo Gaslini 5, 16147 Genoa, Italy; ericaricci@gaslini.org (E.R.); chiara.russo16@icloud.com (C.R.); carolinasaffioti@gaslini.org (C.S.); eliocastagnola@gaslini.org (E.C.); 2 Division of Infectious Diseases, Department of Health Sciences (DISSAL), University of Genoa, 16132 Genoa, Italy; bartalucciclaudia@gmail.com; 3 IRCCS Ospedale Policlinico San Martino, 16132 Genoa, Italy; 4 Department of Neuroscience, Rehabilitation, Ophthalmology, Genetics, Maternal and Child Health (DINOGMI), University of Genova, 16132 Genoa, Italy; 5 Division of Ematology, IRCCS Istituto Giannina Gaslini, 16147 Genoa, Italy; 6 Unit of Bone Marrow Transplantation, IRCCS Istituto Giannina Gaslini, 16147 Genoa, Italy; 7 Division of Neonatal and Pediatric Critical Care and Semi-Intensive Care, IRCCS Istituto Giannina Gaslini, 16147 Genoa, Italy; giacomobrisca@gaslini.org (G.B.); andreamoscatelli@gaslini.org (A.M.); 8 Center for Autoinflammatory Diseases and Immunodeficiencies, IRCCS Istituto Giannina Gaslini, 16147 Genoa, Italy; 9 Hygiene Unit, Department of Health Sciences, Ospedale Policlinico San Martino, University of Genoa, 16132 Genoa, Italy; 10 Divison of Radiology, IRCCS Istituto Giannina Gaslini, 16147 Genoa, Italy; mariabdamasio@gaslini.org; 11 Microbiology Unit, Department of Surgical Sciences and Integrated Diagnostics (DISC), University of Genoa, 16132 Genoa, Italy; anna.marchese@unige.it

**Keywords:** pediatric *Pneumocystis jirovecii* pneumonia, radiological pattern, clinical presentation

## Abstract

Background: *Pneumocytis jirovecii* pneumonia (PJP) has high mortality rates in immunocompromised children, even though routine prophylaxis has decreased in incidence. The aim of this case series is to present the radiological and clinical pathway of PJP in a pediatric population. Description of Cases: All PJP cases in non-HIV/AIDS patients diagnosed at Istituto Giannina Gaslini Pediatric Hospital in Genoa (Italy) from January 2012 until October 2022 were retrospectively evaluated. Nine cases were identified (median age: 8.3 years), and of these, 6/9 underwent prophylaxis with trimethoprim/sulfamethoxazole (TMP/SMX; five once-a-week schedules and one three times-a-week schedule), while 3/9 did not receive this. PJP was diagnosed by real-time PCR for *P. jirovecii*-DNA in respiratory specimens in 7/9 cases and two consecutive positive detections of β-d-glucan (BDG) in the serum in 2/9 cases. Most patients (6/8) had a CT scan with features suggestive of PJP, while one patient did not undergo a scan. All patients were treated with TMP/SMX after a median time from symptoms onset of 3 days. In 7/9 cases, empirical TMP/SMX treatment was initiated after clinical suspicion and radiological evidence and later confirmed by microbiological data. Clinical improvement with the resolution of respiratory failure and 30-day survival included 100% of the study population. Discussion: Due to the difficulty in obtaining biopsy specimens, PJP diagnosis is usually considered probable in most cases. Moreover, the severity of the clinical presentation often leads physicians to start TMP/SMX treatment empirically. BDG proved to be a useful tool for diagnosis, and CT showed good accuracy in identifying typical patterns. In our center, single-day/week prophylaxis was ineffective in high-risk patients; the three-day/week schedule would, therefore, seem preferable and, in any case, should be started promptly in all patients who have an indication of pneumonia.

## 1. Introduction

The incidence of pneumocystis jirovecii pneumonia (PJP) has gradually decreased in the Acquired Immune Deficiency Syndrome (AIDS) population [1], whereas, over the last few decades, it has increased in patients affected by immunocompromising conditions [2], with infection rate ranging from 15 to 45% in the absence of antimicrobial prophylaxis [3]. Conditions associated with a higher risk of PJP are chemotherapy, hematopoietic stem cell transplantation (HCT), severe defects in T-cell immunity, lymphopenia, receiving high doses of corticosteroids for any reasons, and autoimmune diseases in particular [4,5,6,7]. In immunocompromised patients, the introduction of trimethoprim-sulfamethoxazole (TMP/SMX) prophylaxis led to a decline in the rate of PJP infections [4,8,9,10,11]. However, despite the introduction of this highly effective prophylaxis [12,13,14], the PJP-associated mortality rate among children with the non-human immunodeficiency virus (HIV) is still high in developing countries, ranging from 40 to 50% [15,16].

In non-HIV patients, PJP presents as acute and rapidly progressive respiratory deterioration [17,18,19]. A wide variety of radiological features are found both in AIDS- and non-AIDS-associated diseases [20,21], with typical bilateral ground-glass opacities on the upper lobes and peripheral sparing in non-HIV immunocompromised patients [22,23,24,25]. Less common radiological features are nodules, cavities, cystic or honeycomb lesions, pneumothorax, pleural effusion, hilar enlargement, and pneumothorax [23,26,27,28,29]. The gold standard for identifying pulmonary findings is high-resolution computerized tomography (CT) [27,30,31].

The proven diagnosis of PJP relies on the histological identification of trophic and cystic forms of trophozoites. However, since lung biopsy is not always feasible, often, clinicians rely on probable diagnosis, which needs clinical, radiological, and microbiological criteria [12,32], e.g., a polymerase chain reaction (PCR) showing high sensitivity [33,34,35], and serum/plasmatic (1–3)-β-D-glucan (BDG) [36,37,38,39].

This case series aims to describe the typical and suggestive clinical presentation and the most common CT scan features of PJP in an immunocompromised non-AIDS pediatric population.

## 2. Materials and Methods

### 2.1. Study Population

We retrospectively evaluated data from patients under 20 years old who were diagnosed with proven or probable PJP at the IRCCS Istituto Giannina Gaslini Pediatric Hospital in Genoa (Italy) during the period 1 January 2012–31 October 2022. The identification of episodes was based on data extraction from the laboratories that performed a PCR for Pneumocystis jirovecii and BDG, as well as from the DRG (diagnosis-related group) register of our center. These data were then compared with the medical records. Of the extracted results, we selected children with proven or probable PJP diagnosis by analyzing the patients’ clinical records.

### 2.2. Inclusion Criteria

Five inclusion criteria had to be met by each child included in this study as follows: (i) the presence of relevant pulmonary symptoms (i.e., cough or dyspnea), (ii) pulmonary infiltration observed by chest radiography or CT, (iii) the detection of *P. jirovecii* by real-time PCR in respiratory specimens and/or two consecutive positive detections of BDG in the serum, (iv) the ruling out of another possible invasive fungal diseases (IFDs), and (v) the availability of 30-day follow-up.

### 2.3. Diagnosis of PJP

Proven and probable PJP was defined according to the “Revised EORTC/MSGERC Invasive Fungal Disease Definitions” [12], in which “proven PJP” was defined by the presence of the clinical and microbiological criteria of PJP diagnosis plus the demonstration of *P. jirovecii* by microscopy using conventional or immunofluorescence straining on tissue or respiratory specimens; “probable PJP” was defined by the presence of the clinical and microbiological criteria of a PJP diagnosis plus the detection of *P. jirovecii* by real-time PCR in the respiratory specimens or detection of BDG in the serum. Other possible IFDs were ruled out thanks to BAL cultures for molds, serum galactomannan if the patient was neutropenic, and blood cultures.

### 2.4. Data Collection

Clinical data collected included general demographic information, underlying diseases, immunosuppressive therapies, clinical symptomatology, laboratory values, and 30-day hospital mortality. Any further episodes of PJP after the first episode in the subsequent two-year period were also retrospectively analyzed.

Demographical and clinical details were retrieved from the hospital’s computerized record system. For each patient, symptoms from clinical onset to diagnosis were recorded.

### 2.5. Imaging Review

All CT scans were reviewed by a radiologist experienced in pediatric radiology (the author M.B.D), who provided a detailed description of the images for each patient. After a literature review on the typical radiological features of PJP in both the adult and pediatric population, the radiologist classified the CT scans as “typical features associated with PJP” and “non-typical features associated with PJP”.

### 2.6. Statistical Analysis

Continuous variables were described by the median and range or interquartile range (IQR). Categorical variables were described by numbers and percentages.

## 3. Results

### 3.1. Clinical and Epidemiological Characteristics of Study Population

A total of 9 children were included in this study, with a median age of 8.3 years (IQR 3.3–13.9), of which 6/9 (66.6%) were female. They all received a diagnosis of probable PJP, though none of them had the criteria for a proven one. The clinical and epidemiological characteristics of the study population are summarized in Table 1. Steroids (with an equivalent dose of prednisone ≥ 0.3 mg/kg per day for at least 2 weeks before diagnosis) and/or other immunosuppressive/anti-inflammatory drugs were administered to 7/9 patients. In the 3 patients who developed PJP after allogeneic HCT, the infection was diagnosed at a median of 27 days (IQR 26.5–73.5) after the procedure, and, at that time, 2/3 (66.6%) were affected by active graft-versus-host-disease (GvHD) with gastrointestinal involvement.

Five out of nine (55.5%) patients had profound lymphocytopenia with CD4 cell count < 200/mm^3^ at the time of PJP diagnosis.

### 3.2. Study Population

Six patients (66.6%) were receiving TMP/SMX prophylaxis with a once-a-week (*n* = 5) or three-weekly (*n* = 1) regimen and one adolescent patient (patient 5) had poor treatment adherence due to his non-acceptance of the underlying disease and social difficulties.

The remaining three patients did not receive prophylaxis, although they should have since they were all receiving a dose equivalent of prednisone ≥ 0.3 mg/kg per day for at least 2 weeks before PJP diagnosis.

### 3.3. Clinical Presentation

All patients presented with acute respiratory failure, characterized by a rapid respiratory deterioration requiring supplemental oxygen therapy, which in 4/9 cases (44.4%) was represented by high-flow nasal cannula (HFNC) and/or continuous positive airway pressure (CPAP) and in the remaining cases by oxygen at low flows.

### 3.4. PJP Diagnostic Criteria

Microbiological criteria for diagnosis were represented by the positive detection of *P. jirovecii* deoxyribonucleic acid (DNA) by quantitative real-time PCR in respiratory specimens in 4/9 cases (44.4%), qualitative real-time PCR in 3/9 cases (33.3%) and two consecutive positive detections of BDG in serum in 2/9 cases (22.2%). In the study population, 6/9 (66.6%) patients had at least one positive detection of BDG in the serum, with a median value of 486 pg/mL (IQR 405–523); no BDG test was performed on the other three patients. The median time from clinical presentation to microbiological positivity was 2 days (IQR 1–7).

### 3.5. Radiological Features of PJP in the Study Population

Given the acute clinical presentation, all patients received an early radiological examination in a median of 2 days (IQR 0–5) after the onset of respiratory symptoms.

Overall, 8/9 patients (88.8%) underwent a CT scan for a median of 4 days (IQR 0.75–5.75) after symptoms onset. Two patients were unable to perform a CT scan due to clinical severity, which prevented transport to the radiology department for a CT scan. For those two patients, treatment was started based on chest X-ray (CRX) findings, and in one of them, a CT scan was nevertheless performed on day 23 after clinical stabilization to better characterize the pulmonary radiological pattern.

All CT scans were reviewed by an expert pediatric radiologist, who, after a literature review of typical radiological features of PJP in both the adult and pediatric population, classified the CT scans as “typical features associated with PJP” in 6/8 (75%) patients and “non-typical images associated with PJP” in 2/8 (25%) patients.

**Figure 1:**
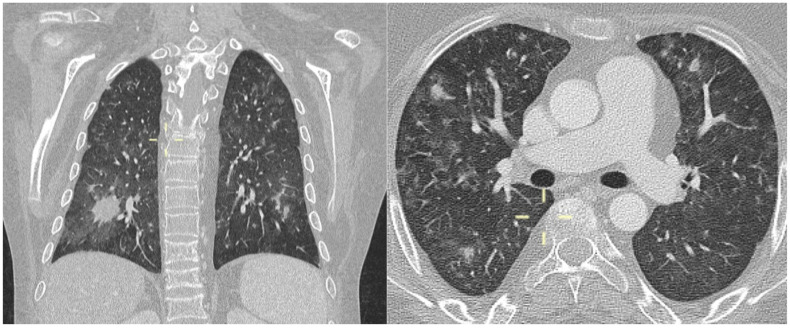
Coronal and axial MPR CT reconstitution (patient 1)—diffuse ground-glass opacities with nodules due to alveolitis with intra-alveolar fibrin and debris. In the coronal view, a definite nodule is evident in the lower right lobe, with ground-glass opacities around the nodule.

**Figure 2:**
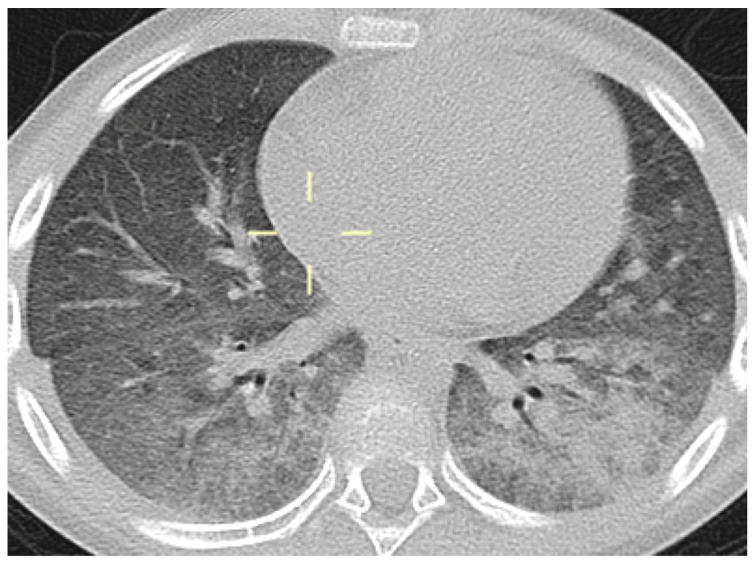
Axial CT reconstitution (patient 2)—bilateral ground-glass opacities, consolidation with air bronchogram, and interstitial thickening with the prevalent involvement of the lower lobes.

**Figure 3:**
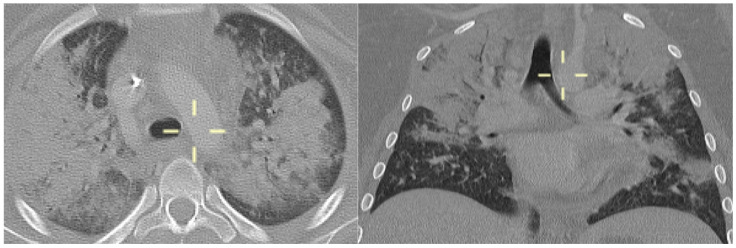
Axial and coronal MPR CT reconstitution (patient 3)—bilateral areas of pulmonary consolidation with air bronchogram with a typical distribution preferentially involving the upper lobes and diffuse interstitial thickening.

**Figure 4:**
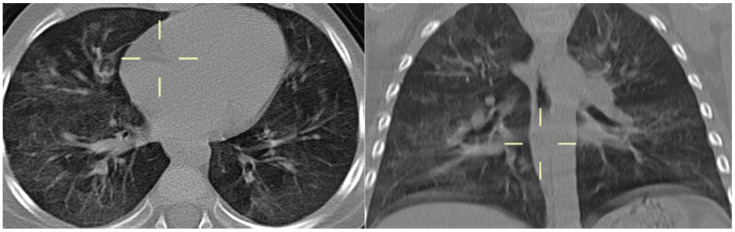
Axial and coronal CT reconstitution (patient 4)—ground-glass opacities with a typical mosaic pattern.

**Figure 5:**
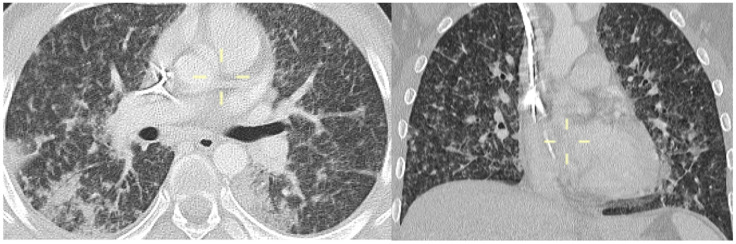
Axial and coronal CT reconstitution (patient 5)—peripheral interstitial thickening with intralobular nodules; subpleural regions are not involved.

**Figure 6:**
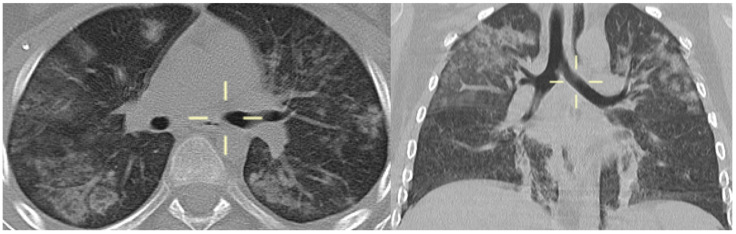
Axial and coronal CT reconstitution (patient 8)—diffuse bilateral alveolar consolidation partly confluent with typical perihilar distribution in the upper lobes.

A detailed description of the TC scans classified as “typical” is presented in Figure 1, Figure 2, Figure 3, Figure 4, Figure 5 and Figure 6.

The two “non-typical images” episodes were both characterized by multiple thickenings, some of which demonstrated air bronchogram spread throughout the entire lung parenchyma without pleural effusion. Multiple thickenings were also observed, some of which had air bronchogram spread throughout the entire lung parenchyma.

### 3.6. Treatment and Outcomes

All patients were treated with a 21-day course of treatment with TMP/SMX, with a median time from symptoms onset to treatment initiation of 3 days (IQR 2–8). In 7/9 (77.7%) cases, TMP-SMX was started empirically based on clinical and radiological findings while awaiting microbiological confirmation; in two cases, TMP-SMX was started based on microbiological positivity, which preceded radiological examination.

Clinical improvement with the resolution of respiratory failure was achieved in all patients, and 30-day survival was achieved for 100% of the study population.

### 3.7. Secondary Prophylaxis

After completing the 21-day treatment cycle, all patients included in the study received secondary prophylaxis with TMP/SMX. Among these, 5/9 patients received three-weekly administration, while 4/9 patients received a single-day course of prophylaxis with once-a-week administration, attributing the failure of prophylaxis to poor compliance rather than to a lack of effectiveness.

In the case of one patient (patient 7) receiving once-weekly prophylaxis, a second episode of PJP occurred 1 year after the previous one. He presented with acute respiratory failure and radiological evidence of interstitial pneumonia, which led to a second course of treatment with TMP/SMX. Unfortunately, the patient died before the completion of therapy due to complications related to the underlying disease.

## 4. Discussion

The study included nine immunocompromised, non-HIV/AIDS children who met specific criteria for probable PJP diagnosis. The study population was at high risk for PJP due to various factors, including immunosuppressive therapies and lymphopenia. The majority of patients were receiving high-dose corticosteroids, which are known to increase the risk of PJP [4,5,6,7]. Indeed, almost half of them were patients with hematological malignancies, confirming the finding that PJP is 1 of the 14 acute toxic effects of antileukemic therapy recognized by the Delphi consensus by 15 international childhood acute lymphoblastic leukemia study groups [40]. It is noteworthy that six patients developed PJP while on prophylaxis with TMP/SMX, with a once-a-week administration for five patients and a three-weekly administration for one patient. Although the effectiveness of once-a-week prophylaxis in the pediatric population has been well described by means of a large epidemiological study [41], failures have been reported in categories of children particularly at risk, such as allogeneic HCT [42]. In these cases, the concomitant presence of GvHD, present in 2/3 of the patients undergoing HCT and under prophylaxis at the time of diagnosis, may have altered the intestinal absorption of TMP/SMX, playing an important role in the failure of prophylaxis. The association between the presence of GvHD and the incidence of PJP is described [43], and this finding made us rethink the type of anti-PJP prophylaxis that should be undertaken in this particular patient setting [42].

Regarding treatment adherence to chronic care in the pediatric population, our data on poor adherence in an adolescent patient highlight how delicate it is to treat patients with daily therapies in this specific age group [44,45] and also for PJP [46].

Another remarkable aspect to consider is incomplete adherence to the criteria for initiating ani-PJP prophylaxis in the study population, as three patients (patients 1, 8, and 9) in our case series developed an infection in the absence of appropriate prophylaxis, even though this was indicated due to the presence of risk factors (all three patients were receiving high-dose corticosteroid therapy).

The clinical presentation of PJP in our series was characterized by acute and severe respiratory failure, necessitating oxygen therapy in all cases. The prompt radiological examinations, including CT scans, aided in the diagnosis and early treatment of PJP. The majority of CT scans (75%) were classified as showing “typical features associated with PJP”, including diffuse bilateral interstitial pulmonary infiltrates [20,21]. Additionally, CT scans revealed ground-glass opacities on the upper lobes with peripheral sparing, which is a common finding in non-HIV immunocompromised patients with PJP [23,26,27,28]. It is important to note that no radiological findings were pathognomonic of PJP. In fact, in two patients, the radiological presentation was not typical, so non-suggestive imaging should not exclude a diagnosis in case of high-level clinical suspicion. However, in the majority of cases, high-resolution CT proved to be the best radiological method for diagnosing PJP. It has been documented that CRX can be initially normal or specific [27,30,31], and therefore, CT scans play a crucial role in the diagnostic assessment of suspected PJP cases, especially in immunosuppressed pediatric patients.

PJP diagnoses were all probable diagnoses according to the EORTC/MSGERC criteria [12], with a *Pneumocystis* PCR and/or BDG appearing positive in all patients. It has been demonstrated that the sensitivity and specificity of PCR on non-invasive respiratory samples (e.g., sputum) are high at 99 and 96%, while on serum, its sensitivity is 77% and the specificity is 90%, though potentially without distinguishing between colonization and infection [38]. In order to reduce possible confounding factors suggestive of colonization rather than infection, in our clinical practice, a *Pneumocystis* PCR was exclusively performed in patients with strong clinical suspicion, predisposing underlying conditions, and/or compatible radiological findings. It is usually performed only on BAL in patients who have undergone bronchoscopy, which, despite being a safe procedure, is an invasive test, particularly for pediatric patients. The role of PCR and serum BDG showed potential as a supportive diagnostic tool, especially when combined with other *P. jirovecii*-specific assays in adults [36,37,38,39], and the combination of these two tests increased both positive and negative predictive values [47]; therefore, the use of BDG for diagnosing PJP in the pediatric population could be useful, while it is generally not recommended for the diagnosis of IFD [48].

Treatment with TMP/SMX was initiated promptly in all patients upon clinical and radiological suspicion, leading to clinical improvement and 100% 30-day survival in the study population.

A remarkable aspect that emerged from the analysis of this case series was the failure of secondary prophylaxis, which became apparent after the first episode during a once-weekly administration schedule of TMP/SMX as secondary prophylaxis. This finding, in addition to the data on the failure of such a scheme of applying for primary prophylaxis [42], should make one rethink the indications of a daily or three-weekly administration in the pediatric population.

In conclusion, PJP is a significant cause of morbidity and mortality in immunocompromised children, and early recognition of this disease is important to enable the rapid initiation of target therapy. In this setting, CT plays a crucial role in recognizing compatible radiological patterns, and combined with molecular detection by PCR and the detection of serum BDG in suspected cases, allows the diagnosis to be confirmed in pediatric patients.

## Figures and Tables

**Table 1:** Clinical and demographic characteristics of study population.

Patient ID	Age (Years)/Sex	Underlining Condition	HCT Type	Time from HCT to PJP (Days)	Ongoing GvHD	Comorbidities at Diagnosis	CS at Diagnosis	Immunosuppressive Drugs at Diagnosis	Ongoing Lympho-penia	Ongoing PJP Prophylaxis Regimen	Outcome
#1	19/M	JIA	No	NA	NA	SARS-CoV-2 pneumonia	Yes	CsA, canakinumab, MAS 825, anakinra, eculizumab	Yes	No	Full recovery
#2	8/F	ALL	No	NA	NA	No	No	MTX, mercaptopurine	No	TMP/SMX 1/w	Full recovery
#3	13/F	ALL	haplo	26	Yes (grade 2)	No	Yes	CT, rituximab	Yes	TMP/SMX 3/w	Full recovery
#4	2/F	MDS	MUD	120	Yes (grade 4)	No	Yes	CsA, ruxolitinib	No	TMP/SMX 1/w	Full recovery
#5	17/M	MPAL	MUD	27	No	BSI *E. faecium*	Yes	CT, dasatinib	Yes	TMP/SMX 1/w	Full recovery
#6	1/F	PI	No	NA	NA	No	Yes	anakinra, sirolimus	Yes	TMP/SMX 1/w	Full recovery
#7	3/M	PI	No	NA	NA	BSI *C. koseri*, esophageal candidiasis	No	No	Yes	TMP/SMX 1/w	Full recovery
#8	14/F	ependymoma	No	NA	NA	No	Yes	CT, RT	No	No	Full recovery
#9	4/F	CD	No	NA	NA	No	Yes	No	No	No	Full recovery

CS: corticosteroids; HCT: hematopoietic stem cell transplantation; GvHD: graft versus host disease; M: male; F: female; JIA: juvenile idiopathic arthritis; ALL: acute lymphoblastic leukemia; MDS: myelodysplastic syndrome; MPAL: mixed-phenotype acute leukemia; PI: primary immunodeficiency; CD: Crohn’s disease; BSI: bloodstream infection; CsA: cyclosporine A; CT: chemotherapy; RT: radiotherapy; MTX: methotrexate; NA: not applicable.

## Data Availability

The data presented in this study are not available for privacy reasons.
